# Longitudinal Associations Between Depressive Symptoms and Quality of Romantic Relationships in Late Adolescence

**DOI:** 10.1007/s10964-021-01511-2

**Published:** 2021-10-18

**Authors:** Daniek H. J. Joosten, Stefanie A. Nelemans, Wim Meeus, Susan Branje

**Affiliations:** Department of Youth and Family, Utrecht University, Utrecht, the Netherlands

**Keywords:** Depressive symptoms, Late adolescence, Romantic relationships, Positive and Negative relationship quality, Longitudinal

## Abstract

While youth with higher levels of depressive symptoms appear to have lower quality romantic relationships, little is known about longitudinal associations for both men and women. Therefore, this study used longitudinal dyadic design to examine both concurrent and longitudinal associations between depressive symptoms and positive as well as negative aspects of romantic relationship quality across two waves one- or two-years apart. The sample consisted of 149 Dutch stable heterosexual couples (149 females and 142 males participated at T_1_) in a stable romantic relationship in late adolescence with a mean age of 20.43 years old at the first wave. Actor-Partner Interdependence models were used to examine potential bidirectional associations over time between depressive symptoms and romantic relationship quality, above and beyond potential concurrent associations and stability of the constructs over time, from the perspective of both romantic partners. Results consistently indicated that men and women who reported higher levels of depressive symptoms perceived less positive aspects (intimacy and support) and more negative aspects (conflict) in their romantic relationship over time. In addition, unexpectedly, when men and women perceived more positive relationship aspects, their partners reported higher levels of depressive symptoms over time. These findings stress that depressive symptoms can interfere with the formation of high-quality romantic relationships.

## Introduction

During late adolescence, romantic partners become increasingly important, to the extent of becoming the primary attachment figure, above and beyond parents and peers (Tillman et al. 2019). Romantic partners come to play a central role in the social lives and emotional experiences in late adolescence. The growing importance of romantic relationships in late adolescence, however, also parallels a rise in depression levels (Rohde et al., 2013). Moreover, low relationship quality during late adolescence has been linked to higher levels of depressive symptoms (e.g., Mirsu-Paun & Oliver 2017). Although studies have shown that youth with higher levels of depressive symptoms have lower quality romantic relationships, longitudinal studies are needed to distinguish between *relationship erosion* effects, in which depressive symptoms deteriorate relationship quality, and *relationship problem* effects, in which lower relationship quality predicts subsequent depressive symptoms. Moreover, dyadic designs are needed to examine whether associations are merely in the eye of the perceiver, or also transcend to the partner in the relationship. Therefore, this study employs a longitudinal dyadic design to examine potential bidirectional associations between both positive and negative aspects of romantic relationships and depressive symptoms over time, above and beyond potential concurrent associations and stability of the constructs over time, from the perspective of both romantic partners.

### Romantic Relationship Quality in Late Adolescence

Across late adolescence and early adulthood, romantic relationships become gradually more meaningful in terms of emotional and physical intimacy and begin to serve an “attachment-function” (Seiffge-Krenke, 2003). According to the *Developmental-Contextual perspective* (Brown, 1999), next to “practical” factors, such as gaining status and fitting in, emotional factors, such as intimacy and support (Moss & Schwebel, 1993), become increasingly important into adolescent’s evaluation of romantic relationships (Brown, 1999). The *Developmental Task perspective* further suggests that romantic relationships during late adolescence and early adulthood are future oriented, (more) stable, salient, and gain psychological value (Meeus et al., 2007). Romantic relationships may, however, also involve negative aspects such as conflict, which includes misunderstandings, disagreements, criticism, and sarcasm or rejection in the communication process. Conflict may arise when partners show opposing actions or have incompatible objectives or ideas (Mackinnon et al., 2012). Having close relationships with romantic partners is important for adolescents’ development, yet “dark sides” of relationships (e.g., conflict) can coexist along with positive aspects of close relationships (e.g., intimacy and support; Sharabi et al., 2016). Therefore, the question arises how the positive and negative aspects of romantic relationships are associated with adolescents’ depressive symptoms, both concurrently and over time.

Some indications herein come from dyadic observational research, which shows that there is a negative association between relationship quality (i.e., commitment, investment) and depressive symptoms among Caucasian heterosexual adolescents. These associations were stronger for women than men (Whitton and Kuryluk 2012). Similarly, Hispanic heterosexual adolescents (i.e., both women and men) who showed more negative interactions in their romantic relationships reported higher levels of depressive symptoms (La Greca and Harrison 2005). Also, negative interactions were positively associated with depressive symptoms in White non-Hispanic, Black, and Hispanic adolescents (Beckmeyer et al. 2018), whereas positive interactions were not significantly associated with depressive symptoms. Furthermore, several studies indicated that, within both homo- and heterosexual Caucasian adolescents, low intimacy and support were associated with higher levels of depressive symptoms (e.g., Daley and Hammen 2002; Williams et al. 2001). Thus, concurrently it seems that depressive symptoms in (late) adolescence and early adulthood are associated with fewer positive aspects (i.e., lower levels of intimacy and support) and more negative aspects (i.e., higher levels of conflict) of romantic relationships.

### Does Relationship Quality Predict Depressive Symptoms Over Time?

Close relationships can have significant costs when relationship quality is low, including depressive symptoms (Sharabi et al., 2016). These costs may occur not only concurrently, but also over time. According to the *Stress and Coping Model* (Davila, 2008), adolescents experience stress when their relationships are of low quality, characterized by few positive aspects such as intimacy and support and many negative aspects such as conflict. Youth who do not have sufficient coping resources are more likely to develop depressive symptoms after such stressful experiences (e.g., Rudolph et al., 2008). Thus, negative experiences that may occur in romantic relationships might set the stage for increased risk to develop depressive symptoms. In addition, according to *IPARTheory’s personality subtheory* (Rohner, 2016), rejection by significant others, including conflict, may lead to a negative worldview and depressed affect. Theoretically, “these dispositions are expected to emerge because of intense psychological pain produced by perceived rejection” (Rohner, 2016, p. 10).

Few studies have investigated longitudinal associations between romantic relationship quality and depressive symptoms. The existing studies suggest that Caucasian heterosexual adolescents who date, particularly those who experience stress in their romantic relationships, report higher levels of depressive symptoms than their non-dating peers over time (Davila et al., 2004). Similarly, it was found that Caucasian heterosexual adults in a romantic relationship identify poor relationship quality (i.e., frequent conflict) as an important predictor for their depressive symptoms over time (Gustavson et al., 2012). Another study among Caucasian adult women did not find that lower positive aspects of romantic relationships (i.e., support) were associated with higher levels of depressive symptoms, or the onset of a Major Depressive Disorder, two years later (Stice et al., 2004). For Dutch heterosexual girls, the expression of both positive and negative emotions in their romantic relationship was positively related to their own depressive symptoms over time. Moreover, boys’ expression of positive and negative emotions in the relationship and girls’ negative emotions were related to boys’ depressive symptoms over time (Ha et al., 2014). However, in the same study relationship satisfaction was unrelated to changes in depressive symptoms (Ha et al., 2014). It thus seems that positive and negative aspects of romantic relationships may have distinct and unique effects on change in depressive symptoms over time. Because findings are mixed in the association between romantic relationships and depressive symptoms for women and men over time, this study separately investigated associations for both men and women longitudinally.

### Do Depressive Symptoms Predict Relationship Quality Over Time?

The opposite direction in the transactional association, in which initial depressive symptoms predict worsening of romantic relationship quality, is also plausible. According to the *Theory of Relational Erosion* (Coyne et al., 1991) and the *Stress Generation Hypothesis* (Hammen, 1991), depressive symptoms generate stress in relationships, which in turn may affect interpersonal behavior, experiences, and choices. Specifically, depressive symptoms may interfere with adaptive interpersonal functioning and over time lead to decreases in the quality of romantic relationships, in the form of fewer positive and more negative aspects in the romantic relationship. Also, the relationship erosion hypothesis would predict that depression precedes poorer quality of the romantic relationship in late adolescence and early adulthood (Joiner et al., 1992).

The few prospective studies that have investigated associations between depressive symptoms and later relationship quality in late adolescence suggest that depressive symptoms may indeed longitudinally predict disruptions in several aspects of romantic relationships. For example, it was found that Western adolescents with higher depressive symptoms experienced fewer positive aspects (e.g., intimacy, support) and more negative aspects (e.g., negative interaction, conflict) in their romantic relationship over time (Rudolph et al., 2008). Also, in Canadian heterosexual college students, depressive symptoms were both an antecedent and a consequence of dyadic conflict for both men and women (Mackinnon et al., 2012). Furthermore, in heterosexual romantic relationships, depressed Caucasian adolescent women as well as their partners perceived these women as less interpersonally competent compared to their non-depressed counterparts (Daley & Hammen, 2002). Also, higher initial levels of depressive symptoms predicted higher levels of relationship conflict, more rejection, and lower support from early adolescence to late adolescence in a representative sample in the United States with both hetero- and homosexual participants (70% Caucasian, 95% heterosexual; Vujeva & Furman, 2011). In addition, depressive symptoms predicted decreases in positive aspects of relationship relationships two years later among adolescent Caucasian women (Stice et al., 2004). Thus, there is some support for the longitudinal association between initial depressive symptoms and later lower romantic relationship quality.

### Dyadic Experiences of Romantic Relationships

Given that romantic partners function within a dyadic system, the understanding of romantic relationships requires the study of the complex dynamic process between partners within this system over time. Because the experience of romantic relationships depends on dyadic interactions that may be perceived differently by the different partners, it is important to assess both partners’ perspectives on the quality of the romantic relationship and their depressive symptoms over time. This allows to examine concurrent and over time associations between one’s own depressive symptoms and one’s own perceptions of relationship quality (i.e., associations within the same dyad member; a so-called *actor effect*) as well as associations between a person’s depressive symptoms and his or her partner’s perception of the quality of the romantic relationship (a so-called *partner effect*; Cook & Kenny, 2005).

The few existing studies that have specifically investigated actor and partner effects showed mixed findings in adolescence and adulthood. For example, in a sample of heterosexual Caucasian adult couples depressive symptoms exerted actor effects, but not partners effects, on relationship quality cross-sectionally for both men and women (Knobloch and Knobloch-Fedders, 2010). Similarly, another study found no evidence of one partner’s romantic relationship satisfaction predicting the other partner’s depressive symptoms over time in an early adult Caucasian sample for both husbands and their wives (Fincham et al., 1997). Another study among Dutch heterosexual adolescents showed consistent actor effects, such that boys and girls with more depressive symptoms indicated that they interacted more negatively and less positively with their partner. In this study, only one partner effect was found, suggesting that girls’ depressive symptoms were related to more positive interaction behavior over time in the perception of boys (Ha et al., 2012). In contrast to previously mentioned studies, there is also some evidence for strong cross-sectional actor and partner effects in the association between depressive symptoms and relationship quality for heterosexual adults (e.g., Whisman et al., 2004).

Furthermore, although the findings reported so far were generally similar for men and women (e.g., Mackinnon et al., 2012, Vujeva & Furman, 2011) some studies suggest there may be gender differences in associations between depressive symptoms and aspects of the romantic relationship. Specifically, previous research in heterosexual couples indicated stronger associations between perceived positive relationship quality and concurrent depressive symptoms among Caucasian adult women than men (Whisman, 2001). Stress generation in romantic relationships has also been found to be stronger among Caucasian adolescent women than men (Davila et al., 1997; Herr et al., 2007) and women are more likely than men to respond to interpersonal stress in ways that exacerbate depressive symptoms, such as ruminating about their problems (Rose & Rudolph, 2006). In addition, it is often assumed that Caucasian adolescent and adult men are less affected by the quality of romantic relationships than women (Levenson et al., 1993). However, most studies have failed to find gender differences in longitudinal associations between perceived relationship quality and depressive symptoms (Kouros et al., 2008; Whisman & Uebelacker, 2009). It is therefore important to examine potential gender-specific associations in heterosexual relationships.

## Current Study

Although studies have shown that youth with more depressive symptoms have lower quality romantic relationships, longitudinal studies are needed to distinguish between *relationship erosion* effects, in which depressive symptoms deteriorate relationship quality, and *relationship problem* effects, in which lower relationship quality predicts subsequent depressive symptoms. Moreover, dyadic designs are needed to distinguish between actor and partner effects in these associations. The current study used a longitudinal dyadic design to examine potential bidirectional associations between positive (i.e., intimacy and support) and negative (i.e., conflict) aspects of romantic relationships and depressive symptoms over time from the perspective of both romantic partners in a stable heterosexual romantic relationship. Longitudinal effects were examined above and beyond potential concurrent associations and stability of the constructs using Actor-Partner-Interdependence Models. Because the experience of romantic relationships is dependent on dyadic interactions that may be perceived differently by both partners, both actor and partner effects were investigated. Furthermore, the focus on heterosexual romantic relationships made it possible to examine potential gender differences in associations. It was hypothesized to find both actor and partner effects in associations. Specifically, it was hypothesized that adolescents’ perceived negative relationship quality (i.e., conflict) was positively associated with their own and their partners’ depressive symptoms, concurrently and bidirectionally over time. In addition, adolescents’ perceived positive relationship quality (i.e., intimacy and support) was expected to be negatively associated with their own and their partners’ depressive symptoms, concurrently and bidirectionally over time. Furthermore, it was hypothesized that the associations were stronger for women than for men.

## Methods

### Procedure

Data were part of the RADAR (Research on Adolescent Development And Relationships) study (Branje and Meeus 2018), an ongoing long-term longitudinal community study that follows two cohorts of adolescents: The “old” cohort that started in 2001 with 239 adolescents (46% male, *M*_age_ T1 = 12.70, *SD*_age_ = 0.41) and the “young” cohort that started in 2005 with 497 Dutch adolescents (57% male, *M*_age_ T1 = 13.03, *SD*_age_ = 0.46). RADAR focuses on adolescent psychosocial development in its broadest sense (e.g., personality, identity, and psychopathology) and relationships with significant others (e.g., parents, siblings, best friends, and intimate partners). From late adolescence onwards, the intimate partner of the adolescents was also invited to participate in the study.

RADAR-Old participants were originally recruited from various secondary schools in the province of Utrecht, the Netherlands. From a large longitudinal sample that was asked to fill out self-report questionnaires (*N* = 923), participants were invited after the first measurement wave to participate in a full family design (*n* = 324). After wave 5, participants from this sample were asked to continue participation in the long-term RADAR study, resulting in 239 participants. RADAR-Old includes nine annual measurement waves across adolescence and early adulthood, followed by biennial waves. This study included data from the first assessments with intimate partner data, which were wave 8 and 9.

RADAR-Young participants were originally recruited from a high number of randomly selected schools in the western and central regions of the Netherlands, resulting in 497 participants (1–3 participants per school). RADAR-Young includes six annual measurement waves across adolescence, followed by biennial waves from late adolescence onwards. This study included data from the first assessments with intimate partner data, which were wave 7 and 8. This study was approved by the Faculty Ethics Review Board of Utrecht University and the Medical Ethical Committee of Utrecht University Medical Centre.

### Participants

The present study combined data from two cohorts of the ongoing longitudinal RADAR study. When participants were around the age of 19, biennial assessments in the RADAR-Young cohort and annual assessments in the RADAR-Old cohort included questions on romantic relationships and romantic partners were also invited to participate. In addition to this first assessment, also data from the consecutive assessment one or two years later was used. At the time of the first assessment, the sample included 605 adolescents. Of these participants, 149 participants were involved in a stable romantic relationship across the two consecutive assessments and were included in the analyses; 250 adolescents did not have a partner in both assessments and these adolescents were therefore excluded from the analyses. Additionally, adolescents who indicated to not have the same partner at both timepoints (*n* = 202) were excluded from the analyses, as for these participants no dyadic over-time effects could be estimated. Finally, homosexual relationships (*n* = 4) were excluded from the analyses, as the analyses focused on romantic relationships in which dyad members could be distinguished by gender.

The final sample included 149 women and 142 men at Time 1 and 146 women and 142 men at Time 2. In the final sample, women were 19.62 years old at Time 1 (*SD*_age_ = 0.87, age range: 15.20–21.90 years) and 20.99 years old at Time 2 (*SD*_age_ = 0.97, age range 16.20 – 23.99 years). The 142 participating men at Time 1 were on average 21.23 years old (*SD*_age_ = 2.25, age range: 17.92–32.70 years) and at Time 2 on average 22.43 years old (*SD*_age_ = 2.15, age range: 18.61–33.70 years). In addition, most participants reported to be Dutch (98.6%). Participants who had a stable relationship did not differ from participants who did not have a romantic partner on any assessment (*n* = 250) on age, *F*(1, 399) = 0.03, *p* = 0.90, and on depressive symptoms on Time 1, *F*(1, 399) = 1.37, *p* = 0.25, but they did differ on depressive symptoms on Time 2, *F*(1, 397) = 3.98, *p* = 0.03. Specifically, adolescents involved in a stable relationship scored significantly lower on depressive symptoms at Time 2 (*M* = 1.59, *SD* = 0.47) than adolescents without a stable relationship (*M* = 1.67, *SD* = 0.47). The final sample and other excluded participants (i.e., adolescents with a partner only at one timepoint and homosexual relationships; *n* = 206), did not differ on age and depressive symptoms on Time 1 and Time 2 (*p*s > 0.24).

### Measures

#### Depressive symptoms

Depressive symptoms were measured with the Dutch adjusted version of the Reynolds Adolescent Depression Scale 2nd Edition (RADS-2; Reynolds, 2000). The RADS-2 is a self-report questionnaire that consists of 30-items of depressive symptoms that a person generally feels, which are measured on a 4-point scale ranging from 1 (*almost never)* to 4 (*usually*). Sample items include “I feel sad” and “I feel like crying”. In a previous study the validity and reliability of this instrument have found to be good (Osman et al., 2010). In this study, internal consistency for the total depression scale was good at the two timepoints, with Cronbach’s alpha ranging from *α* = 0.93 to *α* = 0.97 across waves and partners.

#### Negative Relationship Quality

The Dutch version of the Network of Relationships Inventory (NRI; Furman & Buhrmester, 1985) assessed the perceived level of conflict within the romantic relationship with 6 items. Sample items include “How often do you and your partner argue with each other?” and “How much do you and your partner get upset with or mad at each other”. Respondents rated the items on a 5-point scale ranging from 1 (*barely or never)* to 5 (*a lot*). Cronbach’s alpha reliabilities were good ranging from *α* = 0.79 to *α* = 0.89 across waves and partners.

#### Positive Relationship Quality

The Dutch version of The Triangular Love Scale (TLS; Overbeek et al., 2007) assessed the perceived level of intimacy within the romantic relationship with 7 items. Sample items include “My partner and I tell each other about private thoughts and feelings” and “I can tell everything to my partner”. Respondents rated the items on a 5-point scale ranging from 1 (*not true at all)* to 5 (*very true*). Cronbach’s alpha reliabilities were good ranging from *α* = 0.78 to *α* = 0.87 across waves and partners.

In addition, the Dutch version of the Network of Relationships Inventory (NRI; Furman and Buhrmester 1985) assessed the perceived level of support within the romantic relationship with 8 items. Sample items include “How much does your partner really care about you?” and “How much does your partner appreciate the things you do?”. Respondents rated the items on a 5-point scale ranging from 1 (*barely or never)* to 5 (*a lot*). Cronbach’s alpha reliabilities were good ranging from *α* = 0.76 to *α* = 0.80 across waves and partners. Intimacy and support were averaged to form the construct of positive relationship quality.

### Statistical Analysis

First, preliminary analyses were performed in SPSS IBM 26. Next, Actor-Partner-Interdependence Models (APIMs; Cook & Kenny, 2005) were constructed in M*plus* version 8.4 (Muthén & Muthén, 1998–2020). By using APIMs, actor- and partner-effects in each model could be tested simultaneously. In these APIMs, the longitudinal bidirectional associations between depressive symptoms and negative and positive aspects of romantic relationships were investigated in separate models. Additionally, gender was used to create so-called *distinguishable dyads* (Sadler et al., 2011): Data were reorganized so that one partner was always female and the other partner was always male.

In the analyses, a fully saturated model was used as baseline model, in which step-by-step equivalent comparable concurrent associations and cross-lagged effects between men and women were separately set to be equal in the search for the best-fitting most parsimonious model. All concurrent associations and cross-lagged paths which could be set equal without resulting in a significantly worse fit, were included in the final model.

Maximum Likelihood estimation with standard errors and chi square robust to non-normality (MLR; Satorra & Bentler, 2010) was used in all models. Full Information Maximum Likelihood (FIML; Muthén & Muthén, 1998–2020) was used to handle missing data, estimating all model parameters based on all available data (resulting in *N* = 149 for all analyses). The model fit was tested by means of (a) χ^2^/*df* ratio should be lower than 3, (b) the Comparative Fit Index (CFI) should be equal to or higher than 0.90, (c) the Root Mean Square Error of Approximation (RMSEA) should be equal to or lower than 0.08, (d) and the Standardized Root Mean Residual (SRMR) should be equal to or lower than 0.08 (Bentler, 2007). The comparative fit of the models was tested with Satorra–Bentler scaled chi-square difference tests (ΔSBχ^2^; Satorra & Bentler, 2010).

## Results

### Descriptive Statistics

Means, standard deviations and correlations among study variables for the total sample are reported in Table 1. Women’s and men’s depressive symptoms were significantly negatively correlated with positive relationship quality as perceived by themselves as well as by their partners, and significantly positively correlated with their own perceived negative relationship quality. Depressive symptoms of women and men were significantly correlated at Time 1, *r* = 0.17, but not significantly correlated at Time 2, *p* = 0.19. A Multivariate Analysis of Variance (MANOVA) showed that women and men did not differ on depressive symptoms and perceived quality of the romantic relationship, *F*(12, 145) = 0.89, *p* = 0.48.

**Table 1:** Means, standard deviations, and correlations among study variables

Variable	*N*	*M*	*SD*	1	2	3	4	5	6	7	8	9	10	11
Women														
1. Depressive symptoms T1	149	1.76	0.50	–										
2. Positive relationship aspects T1	143	4.27	0.39	−0.33**	–									
3. Negative relationship aspects T1	143	1.38	0.43	0.29**	0.29**	–								
4. Depressive symptoms T2	145	1.77	0.46	0.66**	−0.25**	0.13	–							
5. Positive relationship aspects T2	146	4.18	0.44	−0.27**	0.56**	−0.23**	−0.29*	–						
6. Negative relationship aspects T2	146	1.45	0.45	0.32**	0.15	0.39**	0.37**	−0.34**	–					
Men														
7. Depressive symptoms T1	142	1.59	0.41	0.17*	−0.13	0.20*	0.03	−0.14	0.18*	–				
8. Positive relationship aspects T1	141	4.15	0.46	−0.19*	0.38**	−0.06	−0.06	0.23**	−0.14	−0.16	–			
9. Negative relationship aspects T1	142	1.41	0.45	0.10	−0.03	0.51**	0.05	−0.03	0.34**	0.16	−0.15	–		
10. Depressive symptoms T2	141	1.56	0.43	0.13	0.04	−0.04	0.05	−0.06	0.15	0.66**	−0.15	0.06	–	
11. Positive relationship aspectsT2	141	4.10	0.50	−0.16	0.28**	−0.12	−0.01	0.33**	−0.27**	−0.33**	0.58**	−0.21*	−0.34**	–
12. Negative relationship aspects T2	142	1.47	0.46	0.16	0.02	0.36**	0.09	−0.10	0.37**	0.16	−0.09	0.46**	0.18*	−0.39**

*Note*. *p < 0.05. **p < 0.01

### Associations between Depressive Symptoms and Negative Relationship Quality

Results of model testing are reported in Table 2. The most parsimonious solution was the model assuming equality between men and women of all concurrent associations and cross-lagged paths over time. This final model showed a good model fit to the data, SBχ^2^(10) = 7.15, CFI = 1.00, RMSEA [90%] = 0.00 [0.00–0.07], SRMR = 0.05. An overview of all associations is shown in Table 3.

**Table 2:** Model difference tests between an initial saturated model and more parsimonious models in the association between depressive symptoms and relationship quality

	Model difference tests			
	Negative relationship aspects		Positive relationship aspects	
	Δχ ^2^ (Δ*df*)^a^	*p* value	Δχ ^2^ (Δ*df*)^a^	*p* value
Cross-lagged effects stable relationships				
*Actor effects*				
Depressive symptoms T1 → Relationship quality T2	0.71	0.40	2.83	0.09
Relationship quality T1 → Depressive symptoms T2	0.93	0.34	0.00	0.96
*Partner effects*				
Relationship quality T1 → Relationship quality T2	0.11	0.74	0.16	0.69
Depressive symptoms T1 → Relationship quality T2	0.05	0.83	0.37	0.54
Depressive symptoms T1 → Depressive symptoms T2	1.10	0.30	1.44	0.23
Relationship quality T1 → Depressive symptoms T2	2.70	0.10	0.70	0.40
Correlations stable relationships				
*Actor effects*				
Depressive symptoms T1 with relationship quality T1	1.24	0.27	2.91	0.09
Depressive symptoms T2 with relationship quality T2	0.27	0.61	0.01	0.90
*Partner effects*				
Depressive symptoms T1 with relationship quality T1	0.12	0.73	1.16	0.28
Depressive symptoms T2 with relationship quality T2	0.43	0.51	0.84	0.36

*Note*. All models are compared to an initial saturated model, SBχ^2^(0) = 0.00, CFI = 1.00, RMSEA [90% CI] = 0.00 [0.00–0.00], SRMR = 0.00.

**Table 3:** Parameter estimates for negative relationship aspects and positive relationship aspects in association with depressive symptoms for stable relationships

	Negative relationship aspects			Positive relationship aspects		
	*B*	*SE*	β/*r*	*b*	*SE*	β/*r*
Cross-lagged effects						
*Actor effects*						
Depressive symptoms T1 → Relationship quality T2	0.16**	0.06	0.14–0.18	−0.15**	0.06	−0.17 to −0.13
Relationship quality T1 → Depressive symptoms T2	−0.00	0.06	−0.00	−0.08	0.05	−0.09 to −0.07
*Partner effects*						
Depressive symptoms T1 → Relationship quality T2	0.06	0.06	0.06	−0.03	0.06	−0.03
Relationship quality T1 → Depressive symptoms T2	−0.09	0.07	−0.09	0.13*	0.05	0.12 – 0.13
Relationship quality T1 → Relationship quality T2	0.16	0.11	0.14–0.18	0.03	0.05	0.02 – 0.03
Depressive symptoms T1 → Depressive symptoms T2	−0.01	0.06	−0.01	−0.01	0.05	−0.01
Stability paths T1 to T2						
Relationship quality women → relationship quality men	0.25**	0.10	0.24	0.53**	0.08	0.47
Depressive symptoms women → depressive symptoms women	0.61**	0.06	0.66	0.62**	0.06	0.65
Relationship quality men → relationship quality women	0.40**	0.10	0.35	0.59**	0.07	0.55
Depressive symptoms men → depressive symptoms men	0.71**	0.09	0.69	0.67**	0.09	0.66
Covariances						
*Time 1*						
*Actor effects*						
Relationship quality with depressive symptoms	0.05**	0.01	0.22–0.24	−0.05**	0.01	−0.25 to −0.24
*Partner effects*						
Relationship quality with depressive symptoms	0.03**	0.01	0.14–0.18	−0.03*	0.01	−0.17 to −0.12
Relationship quality with relationship quality	0.10**	0.02	0.51	0.07**	0.02	0.37
Depressive symptoms with depressive symptoms	0.03	0.02	0.17	0.03	0.02	0.16
* Time 2*						
*Actor effects*						
Relationship quality with depressive symptoms	0.03*	0.02	0.23	−0.02	0.01	−0.17
*Partner effects*						
Relationship quality with depressive symptoms	0.01	0.01	0.08–0.09	0.00	0.01	−0.00
Relationship quality with relationship quality	0.02*	0.02	0.21	0.04**	0.01	0.24
Depressive symptoms with depressive symptoms	0.00	0.01	0.04	0.01	0.01	0.07

*Note*. *p < 0.05. **p < 0.01.

For the concurrent associations at Time 1, in line with our expectations actor effects showed that for both men and women perceived negative relationship quality was positively associated with their own depressive symptoms, *βs* = 0.22–0.24. Furthermore, in line with our expectations partner effects showed that adolescents’ depressive symptoms were positively associated with partners’ perceptions of negative relationship quality, *βs* = 0.14–0.18. Whereas higher levels of perceived negative relationship quality of women were positively associated with perceived negative relationship quality reported by men, *β* = 0.51, the depressive symptoms of the two were not significantly associated, *p* = 0.60. For the concurrent associations at Time 2, actor effects showed that perceived negative relationship quality was positively associated with adolescents’ own depressive symptoms, βs = 0.23. Furthermore, perceived negative relationship quality of both partners was positively associated, *β* = 0.21. All other associations at Time 2 were not significant, *p*s > 0.62.

**Fig. 1:**
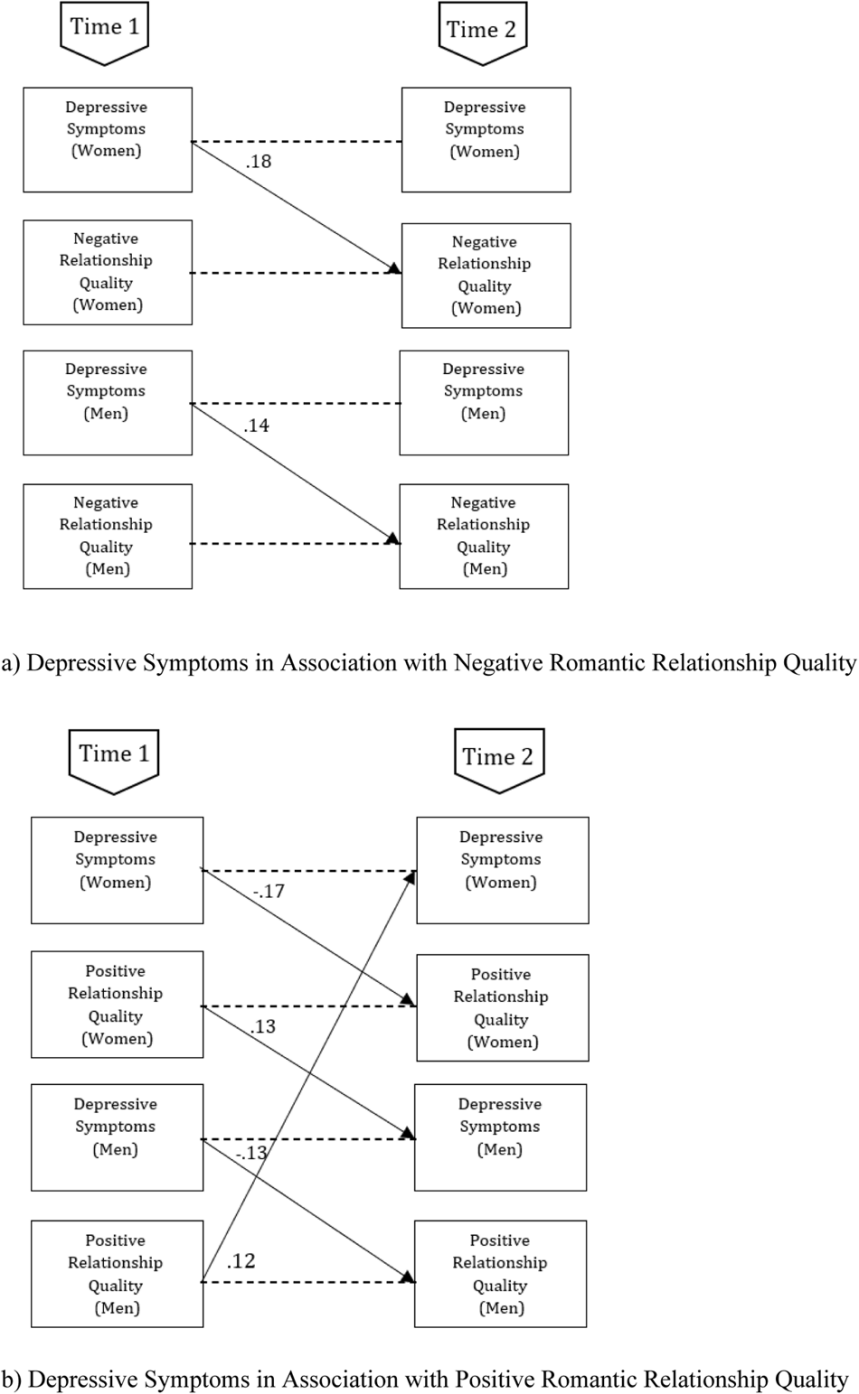
Overview of all significant (*p* < 0.05) Standardized longitudinal associations (β) in the model concerning women’s and men’s depressive symptoms and women’s and men’s reported aspects of relationship quality (**a**) Depressive symptoms in association with negative romantic relationship quality (**b**) Depressive symptoms in association with positive romantic relationship quality

For the longitudinal associations, in contrast to our expectations only significant actor effects from depressive symptoms to relationship quality were found, that is, higher levels of adolescents’ depressive symptoms were longitudinally associated with higher levels of their own perceived negative relationship quality, βs = 0.14–0.18. Also in contrast to our expectations, no significant actor and partner effects were found from perceived negative relationship quality to depressive symptoms over time, *p* > 0.15. Furthermore, adolescents’ depressive symptoms were not associated to their partner’s depressive symptoms over time, *p* > 0.84, nor was partners’ perceived negative relationship quality associated over time, *p* > 0.12. All significant longitudinal associations in the model for negative relationship quality are shown in Fig. 1a.

### Associations between Depressive Symptoms and Positive Relationship Quality

Results of model testing are reported in Table 2. The most parsimonious solution was the model assuming equality between men and women of all concurrent associations and cross-lagged paths in the model. This final model showed a good model fit to the data, SBχ^2^(10) = 8.25, CFI = 1.00, RMSEA [90%] = 0.00 [0.00–0.08], SRMR = 0.05. An overview of all associations is shown in Table 3.

For the concurrent associations at Time 1, in line with our expectations actor effects showed that for both men and women perceived positive relationship quality was negatively associated with their own depressive symptoms, *βs* = −0.25 to −0.24. Furthermore, in line with our expectations partner effects showed that depressive symptoms and perceived positive relationship quality of men and women were negatively associated with each other, *βs* = −0.17 to −0.12. Whereas higher levels of perceived positive relationship quality of women were associated with higher levels of perceived positive relationship quality of men, *β* = 0.37, the depressive symptoms of the two were not associated, *p* = 0.10. For the concurrent associations at Time 2, only perceived positive relationship quality of men was positively associated with perceived positive relationship quality of women, *β* = 0.24.

For the longitudinal associations, in line with our expectations actor effects were found from depressive symptoms to relationship quality, that is, higher levels of adolescents’ depressive symptoms were longitudinally associated with lower levels of own perceived positive relationship quality, βs = −0.17 to −0.13. In line with our expectations also partner effects were found, that is, when adolescents reported higher perceived positive relationship quality, their partner reported more depressive symptoms at a later moment in time, *βs* = 0.12–0.13. However, as the correlations between Time 1 depressive symptoms and Time 2 perceived positive relationship quality of the partner were non-significant or negative and significant (Table 1), this might reflect a suppressor effect. Adolescents’ depressive symptoms were not associated with their partners’ depressive symptoms over time, *p* > 0.80, nor were partners’ perceptions of positive relationship quality associated, *p* > 0.59. All significant longitudinal associations in the model for positive relationship quality are shown in Fig. 1b.

### Sensitivity Analyses

In all final models, age was included as potential covariate by regressing all variables on age. Results suggested that age was statistically associated with women’s perceived positive relationship quality at Time 1, *β* = 0.14, *p* = 0.05, and men’s perceived positive and negative relationship quality at Time 2, *β* = −0.17, *p* = 0.00, and *β* = −0.06, *p* = 0.02, respectively. None of the results was influenced by including age as covariate.

## Discussion

Late adolescence is an important period for forming and maintaining romantic relationships. Yet, this period is also marked by an increase in depressive symptoms. Studies have shown that there are concurrent associations between higher levels of depressive symptoms and lower relationship quality (Mackinnon et al., 2012), but studies into its longitudinal associations and direction of effects are scarce. Longitudinal studies are needed to examine potential relationship erosion effects, in which depressive symptoms deteriorate relationship quality, and relationship problem effects, in which lower relationship quality predicts subsequent depressive symptoms. Moreover, dyadic designs are needed to examine whether associations are merely in the eye of the perceiver, or also transcend to the partner in the relationship. Similarly, gender differences should be examined to investigate whether associations are equally strong for men and women. Therefore, this study aimed to examine (1) concurrent and over-time bidirectional associations between depressive symptoms and both positive and negative aspects of romantic relationship quality, (2) actor and partner effects in these associations by considering perspectives of both romantic partners, and (3) potential gender differences in these associations. These associations were tested in a longitudinal dyadic design using Actor-Partner Interdependence Models.

Results from this study showed that there were both concurrent and over-time associations between depressive symptoms and aspects of romantic relationship quality. None of the associations differed significantly between males and females. Concurrently, lower perceived positive relationship quality and higher perceived negative relationship quality were associated with more depressive symptoms. Longitudinally, depressive symptoms predicted lower levels of self-perceived positive relationship quality and higher levels of self-perceived negative relationship quality over time (i.e., actor effects). Furthermore, higher perceived positive relationship quality by adolescents predicted higher levels of their partners’ subsequent depressive symptoms over time (i.e., partner effects), although this finding may reflect a suppression effect.

### Concurrent Associations

In line with previous research (e.g., La Greca & Harrison, 2005; Williams et al., 2001), the findings showed consistent concurrent associations between relationship quality and depressive symptoms. Romantic relationships during late adolescence may provide an important source of support and contribute in positive ways to the mental health of adolescents in terms of lower levels of depressive symptoms. However, this study indicated that negative experiences in romantic relationships could also contribute to internal distress that may elicit feelings of depression. Especially robust were findings on actor effects in the association between negative relationship quality and depressive symptoms. This may be explained by the fact that depressive symptoms within individuals are also associated with a negative cognitive bias (e.g., Peckham et al., 2010), thereby coloring the perception of people with depressive symptoms more negative in general and their perceptions of their relationship specifically.

### Does Relationship Quality Predict Depressive Symptoms?

The findings from this study consistently indicated that low romantic relationship quality did not significantly predict higher levels of depressive symptoms over time. That is, neither in the perception of men nor women did relationship quality predict depressive symptoms longitudinally. These finding are contradicting the hypothesized stress and coping model and some previous longitudinal findings (e.g., Davila et al., 2004). However, the studies finding support for this association (e.g., Davila, 2008) mainly included adolescent girls. The findings of this study, with an older age group, turned out to be different. In line with the current findings, two other studies have also shown that a lack of positive qualities in a relationship is not directly related to higher levels of depressive symptoms (Prinstein et al., 2005; Stice et al., 2004). However, it could be that lower relationship quality predicts higher levels of depressive symptoms when moderated by other risk factors. For example, it was found that low positive relationship quality interacted with excessive reassurance seeking to predict higher levels of depressive symptoms (Prinstein et al., 2005). Given the lack of support for a main effect of low relationship quality predicting future depressive symptoms, it may be important to investigate moderating factors in the association between depressive symptoms and romantic relationship quality.

Unexpectedly, however, higher perceived positive relationship quality by adolescents predicted more depressive symptoms in their partner over time (i.e., partner effect). Perhaps co-rumination may have played a role in this positive longitudinal association between perceived positive relationship quality and depressive symptoms. Co-rumination, or the excessive discussion of problems within social relationships (Rose, 2002), is positively correlated with positive relationship quality and closeness, but also predicts higher levels of depressive symptoms (Rose et al., 2007). This effect could, however, also be explained by statistical *suppression*, since the correlation between depressive symptoms and partners perceived positive relationship quality was nonsignificant or negative (see Table 1) while the longitudinal regression coefficient was positive. Due to the inclusion of other variables in the statistical model, the predictive validity of adolescents’ perceived positive relationship quality on partners’ depressive symptoms might have increased and changed in sign (Conger, 1974).

### Do Depressive Symptoms Predict Relationship Quality?

For the longitudinal association between depressive symptoms and romantic relationship quality, own depressive symptoms consistently predicted higher levels of own perceived negative relationship quality and lower levels of own perceived positive relationship quality over time. These results are consistent with prior research and theory proposing that characteristics and behaviors associated with depression disrupt interpersonal functioning (Joiner & Coyne, 1999; Rudolph et al., 2008). The findings specifically suggest that depressive symptoms interfere with high-quality romantic relationships, such that adolescents with depressive symptoms have relationships with fewer positive aspects (e.g., intimacy, support) and more negative aspects (e.g., conflict) over time. These findings are also in line with Coyne’s (1976a) interactional theory of depression and a relationship erosion perspective (Branje et al., 2010). These theories suggest that eventually depressive symptoms are assumed to set in motion a process of support erosion in which the interaction becomes increasingly rejecting (e.g., Nelemans et al., 2014). High depressive symptoms may thereby erode the romantic relationship over time.

### Dyadic Experiences of Relationships

In the associations mainly actor effects were found. These findings could potentially be explained by a negative cognitive bias associated with depressive symptoms. A substantial number of studies on emotional information processing in depressed samples provide evidence that depression is characterized by attention, interpretation, and memory biases, especially for negative information (e.g., Peckham et al., 2010). From this, it could be argued that adolescents with higher levels of depressive symptoms also experience their relationship more negatively due to this cognitive bias. Although individuals grappling with depressive symptoms reported less relationship quality for themselves, partner effects did emerge inconsistently.

These findings depart from previous work in which actors’ and partners’ depressive symptoms predicted relationship satisfaction (Whisman et al., 2004), but are in line with other findings also showing no support for partner effects (e.g., Knobloch & Knobloch-Fedders, 2010). On average, the participants of this study were younger and in relationships for shorter time intervals than in studies finding support for partner effects. It is possible that partner depressive symptoms do not have an impact on the overall quality of one’s relationship at this age and on this time-frame. In a recent study, stress experienced by individuals and partners was associated with lower relationship quality for adult couples, but not adolescent couples (ages 16–22; Breitenstein et al., 2018). It may be that adolescents are better able to protect their relationship from daily hassles and to focus on positive aspects of their relationships, as most of them are not cohabitating (yet). Additionally, developmental theories emphasize the self-focused nature of adolescents (Arnett, 2007). Individuals may shift toward emphasizing mutual rather than personal gains when they mature and their relationships become more interdependent (Laursen & Jensen-Campbell, 1999). In fact, in late adolescence a partner may become an attachment figure only after a relatively long relationship duration (Furman & Wehner, 1997). Therefore, partner depressive symptoms may have a stronger effect on relationship functioning at later stages of development. Given that most of the included participants were 19–22 years old, little support for partner effects may have been found. Additionally, people with depressive symptoms interpret their relationships differently than people without depressive symptoms, suggesting that they overestimate negative behaviors and underestimate positive behaviors (Overall & Hammond, 2013). Future research needs to investigate the degree of agreement and accuracy of the different partners in their judgment of depressive symptoms and perceived relationship quality.

There were no gender differences in the longitudinal associations between romantic relationship quality and depression, which is in accordance with earlier findings (Beach et al., 2003; Kouros et al., 2008). However, other research found that depression and poor marital functioning were more strongly associated for women than for men (Herr et al., 2007). Similarly, previous research found depressive symptoms to be associated with concurrent negative behavior towards spouse in women, but not in men, among newlywed couples (Davila et al., 1997). In contrast, this study’s results indicated that among established couples, relationship quality is affected equally regardless of whether a man or woman shows depressive symptoms. These findings contradict previous assumptions that men are less affected than women by the quality of the romantic relationship (Levenson et al., 1993), because changes in both men’s and women’s depressive symptoms were equally strongly predicted by romantic relationship quality.

### Strengths, Limitations, and Directions for Future Research

Despite several strengths (e.g., longitudinal design, multiple informants, examination of both positive and negative relationship quality), the study also had some limitations. For example, even though this study showed evidence for a number of significant cross-lagged actor and partner effects, the insignificance of some other cross-lagged effects might be due to the relatively small sample size and low statistical power to correctly reject a false null hypothesis.

Moreover, in this study only self-reports of depressive symptoms from women and men from the general community were used. The use of self-reports should not be confused with a clinical diagnosis of a psychiatric disorder. It is likely that the severity of depressive symptoms has implications for romantic involvement overall. For example, people with severe depressive symptoms may not have romantic experiences at all (Davila, 2008), which may result in a different type of impairment than that resulting from involvement in romantic activities with subclinical levels of depression. Preliminary findings of this study indeed indicated that adolescents without relationships report more depressive symptoms than adolescents within a relationship. Future research may examine whether the results are the same in a sample with clinical levels of depression.

Further research is also needed to ensure that the current findings were not specific to the demographic profile of the participant sample. Because a homogeneous sample of Dutch middle-class families was used, the sample was not diverse in terms of ethnicity. This may limit the generalizability of findings to families with different ethnic backgrounds. In addition, only heterosexual couples were used, which limits conclusions about homosexual couples. When using a sample with a more diverse relationship constellation, results may turn out to be different. For example, it has been shown that lesbian couples attach more importance to intimacy in the relationship than heterosexual couples (Kurdek, 1998). Thus, it could be that stronger effects are found for positive qualities when more homosexual couples would be included. Furthermore, transgender and gender diverse youth and young adults are at heightened risk for depression and intimate partner violence (Peitzmeier et al., 2020). Therefore, both diverse relationship constellations and diversity in gender might be important topics to investigate. Another limitation is that the study only examined a longitudinal period of one to two years. Future research should explore different time intervals as it is unknown what the optimal time is for studying longitudinal associations between relationship quality and depressive symptoms (Oppenheimer & Hankin, 2011). In addition, effect sizes were relatively small, although this may be reasonable given that effects were controlled for stability in depressive symptoms.

## Conclusion

Late adolescence is an important period for forming and maintaining romantic relationships as well as depressive symptom development, but dyadic studies into longitudinal direction of effects between adolescent depressive symptoms and romantic relationship quality for both men and women are scarce. This longitudinal community study showed that higher levels of depressive symptoms are concurrently and longitudinally associated with less positive and more negative aspects of romantic relationship quality for both males and females. The longitudinal associations were predominantly consistent with a relationship erosion effect in which depressive symptoms predicted lower levels of positive relationship quality and higher levels of negative relationship quality over time. These effects were mainly actor effects, in which one’s depressive symptoms deteriorated one’s own but not the partner’s perception of the quality of the relationship. Although one partner effect emerged in which higher perceived positive relationship quality predicted higher levels of partners’ subsequent depressive symptoms, this finding may reflect a suppression effect. These findings stress that depressive symptoms can interfere with the formation of high-quality romantic relationships, and may have implications for interventions and treatment for heterosexual couples. During treatment for depressive symptoms, adolescents and young adults may benefit from targeting their relational perceptions and attributions. Making use of empirically supported interventions, such as manualized cognitive-behavioral treatments for youth that include a social skills component (e.g., Stark, 1990), or treatments that exclusively target interpersonal issues such as Interpersonal Psychotherapy (IPT; Cuijpers et al. 2011) may be particularly effective. Furthermore, couples’ therapy focuses on both reducing depression and improving relationship quality (Beach et al., 2003), and this study’s findings indicate that such therapy can be relevant regardless of whether a man or a women seeks help for depression or relationship dissatisfaction. The associations between relationship quality and depressive symptoms also have implications for sexual education in high schools, which currently tends to be focused on biological processes rather than psychological aspects of relationships and interactions with romantic partners. This study seems to indicate that psychological aspects of relationships play an important role in mental health of adolescents and young adults and are important to consider in sexual education in addition to biological processes.
